# Be Divergent, Be Green! The Moderating Role of Gender in the Association Between Divergent Thinking and Pro-Environmental Behaviours in Children

**DOI:** 10.3390/children11121497

**Published:** 2024-12-08

**Authors:** Marta Sannino, Elisa Galli, Cristina Zacheo, Marco Giancola

**Affiliations:** 1 Department of Biotechnological and Applied Clinical Sciences, University of L’Aquila, 67100 L’Aquila, Italy; marta.sannino@graduate.univaq.it (M.S.); cristina.zacheo@libero.it (C.Z.); 2 Department of Neuroscience, Imaging and Clinical Sciences, University G. D’Annunzio of Chieti-Pescara, 66100 Chieti, Italy; elisa.galli@phd.unich.it

**Keywords:** childhood, creativity, pro-ecological behaviours, intrapersonal factor, moderation

## Abstract

Background/Objectives: The environmental crisis has begun as a daily challenge for present and forthcoming generations. This scenario highlights the need to adopt many pro-environmental strategies to avoid its adverse consequences. Consequently, it is of paramount importance to comprehend the fundamental psychological and cognitive characteristics that may encourage young children to participate in Pro-Environmental Behaviours (PEBs). Previous research has explored key psychological factors like values, norms, and beliefs influencing children’s pro-environmental behaviours (PEBs), but the impact of cognitive processes is still debated. This research explored the association between divergent thinking (DT) and PEBs, also addressing the potential involvement of gender. Methods: The study involved 348 children (M_age_ = 8.78 years; SD_age_ = 1.79; range age 6–13; 174 girls) who completed a socio-demographic questionnaire, the Pro-Environmental Behaviour Questionnaire, and the Alternative Uses Task (AUT). Results: The statistical analysis indicated that gender moderates the relationship between children’s DT-creativity and PEBs (B = 0.08, SE = 0.04, t = 2.05; 95% CIs [0.0033, 0.1659]), strengthening this association. Conclusions: These findings yielded further evidence on the impact of cognitive processes, such as DT on PEBs, extending the knowledge regarding the critical role of gender in this intricate relationship.

## 1. Introduction

There is widespread evidence that the current environmental crisis has become a problem of global interest. The exploitation of natural resources and productivity growth have negative effects on the environment, such as global warming, climate change, air pollution, natural resource depletion, and loss of biodiversity [1,2,3,4]. New generations of children and youth growing up today will face these serious environmental challenges, partially caused by human behaviours [5,6,7]. Due to the severity of the impact of the environmental crisis for todays and forthcoming generations [8], much psychological research has attempted to identify the primary mechanisms underpinning pro-environmental behaviours (PEBs) [9]. These behaviours encompass a wide array of effective actions aimed at conserving natural resources [10], such as recycling, reuse of resources, and energy-saving practices [11]. Research has suggested different psychological factors as predictors of PEBs, including moral values, attitudes, and norms [12] as well as personality traits [13,14] and cognitive processes [15]. In the context of cognitive processes, previous research has explored the cognitive drivers of adult’s eco-green behaviours, suggesting executive functions (EFs) as a critical factor in explaining individual variance in PEBs. EFs represent a group of cognitive abilities that rely on top-down control processes that are engaged in the planning, organization, and regulation of complex, goal-oriented action [16,17,18]. Due to the crucial role of EFs in self-regulating goal-directed behaviour, it is plausible that they could effectively contribute to translate environmental concerns into actual behaviours aimed at caring the environment [19]. Indeed, EFs are especially triggered mainly in demanding and complex situations [19], such as in case of environmental challenges. Specifically, Langenbach and colleagues [15] have demonstrated that inhibition, working memory, and cognitive flexibility contribute to translate pro-environmental attitudes into planning strategies and actual PEBs [15]. Indeed, cognitive flexibility which allows selective shifting between mental processes, behavioural responses, perspectives and strategies [19], promotes the switching to long-lasting eco-friendly goals and creative strategies helpful to challenge the environmental crisis [20,21].

Interestingly, an additional cognitive process that has gained momentum in the context of pro-environmental practices is divergent thinking (DT). This latter encompasses the ability to produce multiple and creative solutions, break fixed-rules, and be as original as possible [22,23,24,25]. DT is usually defined in terms of fluency (i.e., that involves the number of ideas generated), flexibility (i.e., that entails the quantity of categorical shifts), originality (i.e., that determines the quality of ideas), and elaboration (i.e., the number of details used to embellish or enrich the ideas) [26,27]. DT ability shows early developmental trajectories since the early stages of life. For instance, evidence suggests that in the first year of life children can already think divergently during non-verbal and non-imitative activities [28].

Interestingly DT ability has been identified as a trigger for different types of environmentally responsible practices, including (1) eco-management (i.e., the physical or direct actions that preserve the environment); (2) consumer and economic action; (3) interpersonal and public persuasion, and (4) political action [29,30]. Similarly, other studies have shown that creativity along with open-mindedness and curiosity represents a key component of sustainable behaviours as well as sustainable lifestyle [29,31].

This evidence has been also confirmed in different developmental stages, such as childhood and adolescence. Indeed, some studies stressed the view that DT was associated with connectedness to nature, strong original self-efficacy and innovative thinking style, also showing that nature engagement may, in turn, promote the creation of original and unconventional ideas [32]. Promoting DT during childhood may help to develop life-skills that are useful for creating alternative strategies and expanding existing ideas [33,34]. In addition, Giancola and colleagues [14] indicated that adolescents’ DT positively affects the disposition toward practices aimed at safeguarding the natural environment. The authors suggested that DT depicts a human virtue that could promote self-creating lifestyles and creative environmental solutions, also allowing for solving problems that involve environmental challenges.

Despite this evidence, the mechanisms involved in the association between children’s DT and PEBs remains under explored thus far. Indeed, this link could be influenced by different cognitive, emotional, and socio-cultural mechanisms including cognitive styles, emotional competences, self-perception, or family background [14]. In this context, biological factors such as gender may be also involved. Indeed, some studies suggested that gender is critical in children’s ability to produce divergent ideas highlighting that boys outperform girls in creativity [35]. Other studies confirmed this evidence in young adults, demonstrating a greater male variability in the figural domain than the verbal domain of DT [36]. This evidence corroborates the widespread greater male variability in creativity (GMVC) hypothesis which suggests that men vary more than women in creativity skills [37]. Moreover, Sandhiya and Bhuvaneswari [38] assumed that male adolescent students perform better in creative tasks and have a higher DT than female students. In a sample of young children Giancola and colleagues [39] have demonstrated that the association between field dependent-independent cognitive style (FDI) and the ability to think divergently was stronger among boys, stressing the critical effect of gender differences in children DT. Overall, these findings support the hypothesis that male gender plays a key role in explaining variability in DT skills from the early developmental stages of life.

Notably, gender differences were also found in PEBs [7,40]. For instance, in a sample of 9- to 12-year-old children, Duron-Ramos and colleagues [40] have demonstrated that the association between connection to nature and PEB is stronger for girls than boys, strengthening the link between females’ emotional connection to nature elements and their “green” behaviours. The reason for this result may be that girls are “normally” and culturally socialised to show helpful attitudes towards others and more altruistic values [41,42]. These findings have been also confirmed in adolescents and young adults [43]. Some studies indicated that females are more likely than males to be involved in green activities [44,45] while others demonstrated higher levels of emotional connectedness to nature [46], pro-ecological attitudes, and empathic connection to nature that evolve in more frequent PEBs [43,46].

Although gender differences may affect both DT and PEBs, it is plausible that boys leverage their DT ability to approach environmental issues more effectively and flexibly than girls. By using their divergence of thinking, boys may better understand the nature and the complexity of environmental issues, allowing them to adopt a more holistic and adaptive approach and act accordingly [14]. This notion implies that DT, especially for boys, can serve as a critical individual cognitive resource that not only fosters the generation of multiple and original solutions, but also a promoting factor for a more sustainable lifestyle [29,47,48]. In other words, while girls’ higher emotional and empathic connection with nature drives their PEBs, for boys’ DT skills feed the individual disposition in engaging ecological strategies and eco-green actions. Accordingly, the research hypothesis has been advanced as follows: gender moderates the relationship between DT and PEBs, with boys showing a stronger association between DT and PEBs than girls.

## 2. Materials and Methods

### 2.1. Participants and Procedure

In the present research, an a priori power analysis through G*Power 3.1 [49] has been conducted to identify an appropriate minimum sample size. The following parameters have been employed: test family: “*F* test analysis”, statistical test: “Linear multiple regression: fixed model, *R*^2^ deviation from zero”, type of analysis: “A priori: Compute required sample size—given α, power and effect size”, α err prob = 0.05, power (1-β err prob) = 0.95, mean effect size *f*^2^ = 0.15 (medium effect), and a maximum number of predictors = 3. The power analysis suggested a recommended minimum sample size of 119. A total of 348 children participated in the study, with an average age of 8.78 years (SD_age_ = 1.79; range_age_ = 6–13). This included 174 boys (M_age_ = 8.84, SD_age_ = 1.76) and 174 girls (M_age_ = 8.72, SD_age_ = 1.81).

The study is a part of a research project about sustainable behaviours in children and was carried out in an Italian primary school located in Ascoli Piceno (Marche, Italy). Data were gathered during December 2022 and the experiment involved the following three phases: (1) researchers informed the school principal about the nature, goals, and main procedures of the study; (2) after the school principal provided her consent, parents/legal guardians received a consent note about the topic, the main aims and assessment of the research. The consent notes also guaranteed anonymity for all participants, specifying that the participation was voluntary; and (3) after obtaining written consent, researchers administered a brief battery of questionnaires to parents/legal guardians, in which socio-demographic information and city area of residence were requested. Children who were authorised by their parents/legal guardians, were asked to complete a short demographic questionnaire, the GEB, and the AUT in the presence of their teacher and a trained research assistant (C.Z.). The study has been conducted in accordance with the Declaration of Helsinki and approved by the Research Ethics Committee of the University of L’Aquila on 27 September 2022.

### 2.2. Measures

#### 2.2.1. Socio-Demographics

A brief socio-demographic questionnaire was used to evaluate children’s age and gender.

#### 2.2.2. Divergent Thinking

The Alternative Uses Task (AUT) from the Torrance Test of Creative Thinking—TTCT Form A [50] involves generating as many alternatives uses for carton boxes as possible within the time limit of 10 min. Although the TTCT comprises a variety of visual and verbal tasks, this research has employed a single-item approach, focusing only on the AUT. This method aims to reduce children’s fatigue, cognitive overload, and variations in attention, thereby ensuring the elicitation of a wide array of responses. Instead of employing the standard criteria defined in the technical manual [50], children’s alternative uses have been evaluated by two trained judges (two females: M_age_ = 24; SD_age_ = 1.41) in terms of fluency (number of appropriate and alternative ideas) and creativity (the degree of uncommonness, remoteness, and cleverness of ideas). Specifically, Creativity was evaluated through the snapshot scoring method [51], which consists of assigning a holistic score of the entire set of alternative uses by weighting uncommonness, remoteness and cleverness on a 5-point Likert-type scale ranging from 1 (not at all creative) to 5 (extremely creative). This method allows for capturing more deeply the multifaceted nature of creativity, focusing not only on the degree of infrequency of the ideas (uncommonness or originality), but also on the extent to which the idea generated is remotely related to everyday objects (remoteness), and the level of irony, humour and brightness of the ideas (cleverness) [51]. The inter-rater correlation for fluency and creativity was α = 0.99 and α = 0.98, respectively.

#### 2.2.3. Pro-Environmental Behaviours

The Pro-Environmental Behaviour Questionnaire (PEBQ) [52] is a short tool adapted from the General Ecological Behaviour Scale by Kaiser and Wilson [53]. The PEBQ has been developed to evaluate children’s PEBs (e.g., reuse, recycling, and conserving resources) within the family context trough of 23 items anchored on a 5-point Likert-type scale ranging from 1 (never) to 5 (always). In the current study, the PEBQ was administered in Italian, based on a prior Italian version of the questionnaire designed for children [54]. The PEBQ showed good psychometric properties, with an acceptable internal consistency (Cronbach’s α = 0.73), confirming its reliability in evaluating PEBs in children.

### 2.3. Statistics

Data were analysed using SPSS Statistics version 24 for Windows (IBM Corporation, Armonk, New York, NY, USA). Data screening was performed through the normality test, z-test, and Harman’s single factor, while to verify preliminarily the relationships among the study variables bivariate correlations were computed. In order to test the potential moderating effect of gender in the association between DT and PEBs, the PROCESS macro for SPSS was used [55]. The significance of the moderating effect was analysed using 5000 resample of bootstrapped estimates with 95% bias-corrected confidence intervals—CIs. The 95% CIs must not cross zero to satisfy the criteria of moderation [56]. All significance was set to *p* < 0.05.

## 3. Results

Data distribution test revealed that all study variables were not normally distributed, while the z-test with ±4.0 z-scores as cutoff [57] indicated no univariate outliers. Moreover, Harman’s single-factor test [58] showed that the variance explained by a single-factor exploratory model was 36.60, revealing no common method bias problems (test critical threshold ≥ 50%). Table 1 reports the correlation among all study variables.

Additionally, the moderation analysis was computed entering one by one DT-fluency and DT-creativity as the independent variable, PEBs as the dependent variable, gender as the moderator, and age as the covariate. As shown in Table 2, results indicated that using DT-fluency as the independent variable, PEBs as the dependent variable, and gender as the moderator (Model 1), we found a non-significant interaction between DT-fluency and gender (*B* = 0.02, *SE* = 0.03, *t* = 0.57; 95% CIs [–0.0429, 0.0782]). Conversely, the analysis showed that with DT-creativity as the independent variable, PEBs as the dependent variable, and gender as the moderator (Model 2), the interaction between DT-creativity and gender was significant (*B* = 0.08, *SE* = 0.04, *t* = 2.05; 95% CIs [0.0033, 0.1659]): DT-creativity predicted PEBs for boys (*B* = 0.07, *SE* = 0.03, *t* = 2.58; 95% CIs [0.0168, 0.1255]) but not for girls (*B* = −0.01, *SE* = 0.03, *t* = −0.43; 95% CIs [−0.0746, 0.0477]).

## 4. Discussion

The current research builds on the evidence that DT plays a key role in explaining individual variance in PEBs [14,15]. DT skills provide the ability to generate flexible and creative solutions [59,60,61,62] and the development of these abilities in the context of early childhood is particularly relevant to understand how children represent the world around them and its environmental challenges [63].

Indeed, DT skills can explain individual differences in creative environmental problem solving and production of original ecological strategies and eco-green solutions [29,64]. Through the dimensions of fluency, flexibility and originality, think divergently can contribute to face situations of uncertainty and pursue sustainable actions and lifestyles [63]. Accordingly, the present study aims to investigate the involvement of gender as a moderator in the association between DT and PEBs in a sample of school-age children.

Results confirmed the research hypothesis, indicating that male gender moderates the relationship between children’s DT and PEBs. These findings align with previous research stressing the key role of the abilities to think originally, being less conformist, and approaching a problem from different points of view in creative green solutions as well as general PEBs [29,38,64]. Particularly, previous studies showed that males exhibit greater variability than females in creativity and unconventional thinking [36], a higher variability of original thinking and unconventionality, and appear to be less conformist [65]. Probably, these results could be interpreted considering the role of DT as a cognitive resource which allows one to face a problem from different points of view and thus to understand its complexity and importance. This cognitive tendency, that is more frequent in males, could lead them to a more flexible and harmonious understanding of environmental issues and all its more complex aspects, thereby enhancing their propensity for PEBs. For boys, it seems that DT skills enhance a predisposition to approach a problem by understanding its full complexity, serving as a promoting factor for a more sustainable lifestyle. Furthermore, the moderation effect related to male gender provided by our study, could be interpreted considering also the educational and socio-cultural context in which today’s children are placed. Indeed, from early childhood girls tend to show less interest and feel less confident in logical-mathematical and scientific subjects [66]. According to the recent STEAM educational approach (Science, Technology, Engineering, Art, and Mathematics), children need to break down different types of problems and foster the development of critical, analytical, and creative thinking [67]. Children’s critical thinking skills reflect the ability to observe a problem from different perspectives, to think flexibly and to be curious [67]. Interestingly, all these abilities that are close to DT skills and male gender, could translate into desire to approach the environment issue in an active and holistic manner and to find green solutions. Overall, these gender differences could be related to cultural stereotypes, moral and social differences linked to gender [41,42,68], but also to varieties in cognitive styles [69] and processes, and to educational level [70]. Indeed, although girls also exhibit DT skills, their pro-environmental actions would seem to be more influenced by empathy or a sense of collective responsibility, probably linked to gender-related socio-cultural expectations [41,42,68].

The present study shows several theoretical and practical implications. Given that DT skills predict individual dispositions towards eco-sustainable actions and emerge from early childhood [28,62,71,72,73,74], it could be helpful to plan workshops and training on DT in the school context. Moreover, training children to think flexibly may also help them to develop critical and analytical thinking and new strategies to face problems around them. Furthermore, DT-centred training with the recent STEAM educational approach could support girls to experiment with creative problem-solving activities and contribute to the development of caring and active “environmental problem-solvers”. Promoting DT during childhood may help to develop life-skills and a set of abilities that are crucial for a positive development in terms of school, academic and everyday life success [29]. Additionally, this research suggested the importance of considering educational and civic programs based on sustainable lifestyles, that could be included in school curricula and promote a positive development of eco-green behaviours from early childhood [75]. Particularly, these programs could be designed to improve students’ understanding of serious environmental problems and their impact on society and humanity. Moreover, these educational programs should aim at teaching children to understand the moral and civic implications of their actions on the natural environment [76,77,78,79,80], encouraging a more active and engaged learning process. Furthermore, family and peer involvement activities may extend the impact of school programs beyond the classroom, promoting a community-wide culture of sustainability.

Despite these theoretical and educational implications, the current study shows some limitations that suggest future research directions. First, this research was cross-sectional and did not provide evidence of causal inferences. Future research should confirm the moderating role of gender using a longitudinal design, as well as to better explore the mechanisms supporting PEBs from childhood to adulthood. Second, in the present study, data has been gathered from one region of Italy, and this may negatively affect the generalizability of the findings. Future research should replicate our results by including a more representative sample.

Third, in the current study, only DT has been considered. To provide a more exhaustive evaluation of the impact of cognitive processes on PEBs, future studies should also consider other aspects of the cognitive sphere, such as executive functioning. Fourth, these findings are mainly based on self-report questionnaires that do not assess real-life PEBs; to overcome this limitation, future research should confirm the study’s hypotheses through objective and simple real-life measures that are more suitable for children [81], possibly to be implemented directly in children’s educational environment. Finally, future research could also investigate the involvement of other psychological factors in the relationship between children’s DT and PEBs, including emotional skills, personality traits (e.g., HEXACO, Dark Tetrad, Trait emotional intelligence), thinking styles, and temperament traits [82,83,84,85,86,87].

In conclusion, the present study provides a more comprehensive view of the main individual factors involved in children’s PEBs, also extending the current knowledge regarding the critical role of gender and offering useful tips for practical interventions to foster environmental education and awareness in children.

## Figures and Tables

**Table 1:** Descriptive statistics and correlations among the study variables divided by gender.

Boys (*N* = 174)							Girls (*N* = 174)					
	Mean	SD	1.	2.	3.	4.	Mean	SD	1.	2.	3.	4.
1. Age	8.84	1.76	1				8.72	1.81	1			
2. DT-fluency	1.94	1.71	0.20 **	1			1.79	1.33	0.18 *	1		
3. DT-creativity	1.82	1.17	0.21 **	0.78 **	1		1.85	1.05	0.22 **	0.69 **	1	
4. PEBs	3.63	0.44	0.14	0.22 **	0.23 **	1	3.58	0.41	0.07	0.00	−0.06	1

Note. DT = Divergent thinking, PEBs = Pro-environmental behaviours. ** *p* < 0.01 (two tailed), * *p* < 0.05 (two tailed).

**Table 2:** Coefficients for the moderating models.

	*B*	*SE*	*t*	LLCI	ULCI
Model 1					
DT-fluency	0.02	0.02	0.71	−0.305	0.0652
Gender	0.01	0.07	0.14	−0.1332	0.1543
DT-fluency × gender	0.02	0.03	0.57	−0.0429	0.0782
Age	0.01	0.01	0.65	−0.0169	0.036
Model 2					
DT-creativity	−0.01	0.03	−0.43	−0.0746	0.0477
Gender	−0.11	0.09	−1.21	−0.2809	−0.0674
DT-creativity × gender	0.08	0.04	2.04	0.0033	0.1659
Age	0.01	0.01	0.06	−0.0176	0.0332

Note. *N* = 348. SE = Standard Error, LLCI = Lower Limit of the 95% Confidence Interval, ULCI = Upper Limit of the 95% Confidence Interval.

## Data Availability

The data presented in this study are available on request from the corresponding author. The data are not publicly available due to privacy reasons.
